# Assessment of Dietary Intake of Long-Distance Race Car Drivers—A Pilot Study

**DOI:** 10.3390/sports6040118

**Published:** 2018-10-12

**Authors:** Edem Korkor Appiah-Dwomoh, Anja Carlsohn, Frank Mayer

**Affiliations:** 1 Clinical Exercise Science, Department of Sports and Health Science, University Outpatient Clinic Potsdam, University of Potsdam, 14469 Potsdam, Brandenburg, Germany; 2 Department of Health Sciences, University of Education Schwaebisch Gmuend, 73525 Schwaebisch Gmuend, Germany; anja.carlsohn@haw-hamburg.de; 3 Center of Sports Medicine, University Outpatient Clinic Potsdam, University of Potsdam, 14469 Potsdam, Brandenburg, Germany; fmayer@uni-potsdam.de; 4 Department of Ecotrophology, Faculty of Life Sciences, University of Applied Science Hamburg, Ulmenliet 20, 21033 Hamburg, Germany

**Keywords:** long-distance race car driving, dietary intake, 24 h recall, pilot study

## Abstract

Long-distance race car drivers are classified as athletes. The sport is physically and mentally demanding, requiring long hours of practice. Therefore, optimal dietary intake is essential for health and performance of the athlete. The aim of the study was to evaluate dietary intake and to compare the data with dietary recommendations for athletes and for the general adult population according to the German Nutrition Society (DGE). A 24-h dietary recall during a competition preparation phase was obtained from 16 male race car drivers (28.3 ± 6.1 years, body mass index (BMI) of 22.9 ± 2.3 kg/m^2^). The mean intake of energy, nutrients, water and alcohol was recorded. The mean energy, vitamin B_2_, vitamin E, folate, fiber, calcium, water and alcohol intake were 2124 ± 814 kcal/day, 1.3 ± 0.5 mg/day, 12.5 ± 9.5 mg/day, 231.0 ± 90.9 ug/day, 21.4 ± 9.4 g/day, 1104 ± 764 mg/day, 3309 ± 1522 mL/day and 0.8 ± 2.5 mL/day respectively. Our study indicated that many of the nutrients studied, including energy and carbohydrate, were below the recommended dietary intake for both athletes and the DGE.

## 1. Introduction

There is supporting evidence linking optimal nutrition to enhanced performance and recovery after exercise [1]. Through nutritional assessment, the nutritional status of the athlete, as well as potential nutritional problems, can be identified, training-induced adaptations can be enhanced, and peak performance can be supported [1]. According to Thomas et al. [2], an appropriate energy intake supports optimal body function and assists in changing body composition. During times of high physical activity, energy, macro- and micronutrient needs must be met to maintain body weight, replenish glycogen stores, and provide adequate protein to build and repair tissues as well as to avoid low energy availability [3]. In addition to these, an increase in the proportion of one macronutrient will lead to a decrease in the proportion of the other macronutrients given energy intake. This will in turn affect the intake of micronutrients [2].

Long-distance race car drivers are classified as athletes [4]. They are skilled in race car driving, possess physical strength, agility, endurance, and participate in competitions [4]. The sport is physically demanding, exposing the athletes to vibration, high temperatures, and long driving hours [5] especially in 24 h competitions (e.g., “Le Mans”). The drivers race 8 h in 24 h competitions with a single lap being 15 turns and 450 braking actions [5]. In training sessions, the drivers are required to drive one fast lap [5]. The physical demands and physiological stresses on the drivers can be compared to those placed on other athletes such as football, basketball, and baseball players [6]. They have similar VO_2_ max values (42.0 to 59.7 mL·kg^−1^·min^−1^) [6] and physical activity ratio (4.92 ± 0.50 to 5.43 ± 0.47 Mets) [7] requiring the development of diverse physical abilities [5] and optimal fitness. To be optimally fit requires a multidisciplinary approach including nutritional considerations. This must fit the motorsport requirements like maintaining low body weight without low energy availability, maintaining energy supply during races, and restoring all that is needed in between races.

In published literature, dietary intake data are provided for athletes in some sport disciplines such as strength sports, team and endurance sports [8,9,10]. However, to the best of our knowledge, no published literature is available on energy and nutrient intake in race car drivers although adequate nutrition is essential for athletes to meet energy demands for training, competition and to optimize performance [11]. According to the German Nutrition Society (DGE), recommended dietary intake values are supposed to apply to 98% of healthy people in Germany [12]. Recommendations intend to help individuals improve and maintain overall health. There are no dietary recommendations for most micronutrients for athletes in general and race car drivers to be specific. However, micronutrient intake recommendations for the general population could cover the needs of athletes [13]. Therefore, one might assume that the race car drivers will at least meet the dietary recommendations for the general population of Germany although it is not guaranteed that the supply will be enough.

Therefore, the aim of the study is to evaluate mean energy and nutrient intake in long-distance race car drivers and to compare it to the dietary recommendations for athletes and for the general adult population according to the DGE. 

## 2. Materials and Methods

A total of 16 male professional long-distance race car drivers were involved in the pilot study. The athletes were preparing for the main competition of the year (24 h race in Le Mans). The subjects were recruited at the Olympic Medical Center of the Outpatient Clinic of the University of Potsdam where they had reported for general examination for a competition preparation phase. All subjects gave a written informed consent to participate in the study. The assessment of anthropometrics and dietary intake was carried out by professional healthcare personnel. The study protocol was approved by the ethics committee of the University of Potsdam. 

Anthropometric data (age, height and body weight) of 16 professional race car drivers were measured with the subjects standing in an upright position [14]. A stable stadiometer and a weighing scale were used for height and body weight measurement respectively. Body-mass index (BMI) was also calculated using the weight in kilograms divided by the height in meters squared. The measurements were carried out with minimal clothes on and without shoes. This was done to describe the characteristics of our subjects and to assess any changes in body mass during the period of the last annual health check.

Dietary intake was recorded using a 24-h dietary recall structured questionnaire (Appendix A). This was used to capture detailed information about all foods and drinks consumed by the subjects during the last 24 h. Portion sizes and the time of meal or snack intake in the previous 24 h were also recorded. The interview was conducted in a single face-to-face interview by the same investigator. The interview lasted approximately 30 min. Nutrient intake was analyzed based on the German food database using PRODI expert software (software version 5.7, PRODI NutriScience, Hausach, Germany). 

Statistical analysis was done descriptively and means and standard deviations (mean ± SD) are provided. 

## 3. Results

The mean age of the drivers was 28.3 ± 6.1 years, they weighed 72.4 ± 7.0 kg, were 178.3 ± 6.5 cm tall, and had a BMI of 22.9 ± 2.3 kg/m^2^. The mean energy, carbohydrate, fat and protein intake were 2124 ± 814 kcal/day, 236.1 ± 96.7 g/day, 95.1 ± 39.4 g/day, 85.6 ± 47.1 g/day respectively. Sodium intake was 2790 ± 1721 mg/day whilst water was 3309 ± 1522 mL/day. Other nutrients like vitamin C, folate and magnesium intake recorded are 161.5 ± 113.0 mg/day, 231.0 ± 90.9 mg/day and 471 ± 335 mg/day, respectively. Table 1 shows the full list of nutrients studied. 

## 4. Discussion

The study aimed to evaluate the mean energy and nutrient intake in long-distance race car drivers and to compare it to the dietary recommendations for athletes and the general adult population in Germany. The present results show that energy, carbohydrate, vitamin B_2_, vitamin E, vitamin D, folate and fiber intake did not meet the recommendation for athletes and the DGE. Protein intake was within the recommended levels for athletes but exceeded that of the DGE. Fat and calcium intake were below and above the dietary recommendation for athletes and DGE respectively. Vitamin B_1_, vitamin B_6_, vitamin B_12_, vitamin C, sodium, magnesium, iron, zinc and water intake were above the recommended dietary intake for the DGE. 

Adequate energy intake is essential for optimal performance in sport [15]. This is the bedrock of the athletes’ diet since it supports calorific expenditure, promotes the maintenance or improvement of strength, endurance and muscle mass [12]. In the current study, the energy intake of the long-distance race car drivers is inadequate. The majority did not meet recommendations of energy for athletes [15] and the general adult population in Germany with a physical activity level (PAL) of 1.8 (3000 kcal/day) [18]. Published literature analyzing the dietary intake of athletes report optimal and inadequate energy intake for different sports disciplines depending on whether intake was recorded during the sporting activity or not [23,24,25]. In the current study, dietary assessment was carried out during a regular health check when athletes were preparing for competition. Therefore, one would assume energy intake would be appropriate because the stress associated with dietary intake during competition is eliminated [26]. However, most of the athletes did not reach the recommended energy intake for athletes [15] and the DGE [18]. The results could have been affected by a change in diet associated with travelling to the venue of measurement, which was different from the normal environment of the athletes. In addition to this, the low energy intake might have been due to under reporting and or forgetting snacks and sugar-rich drinks consumed during the interview period. Sport-specific energy requirements vary greatly between sports [15]. Endurance athletes weighing 50–100 kg are recommended to consume 2500–8000 calories per day whilst strength and power athletes need 44–50 kcal/kg/day of energy intake for optimal performance [15]. To the best of our knowledge there is no published literature on the dietary intake of long-distance race car drivers. Therefore, energy intake must at least reach the guiding values for the DGE with the aim of meeting dietary recommendations for elite athletes in football, basketball and baseball [6]. In addition to this, individual energy needs, and goals must be determined based on age, height, and weight of the athlete [15]. 

The mean carbohydrate intake of 3.3 ± 1.5 g/kg/day recorded in the current study was lower than recommended and lower than that reported for endurance, team sports and strength athletes (4.0–6.8 g/kg) [24,27]. The result could have been affected by under-reporting or limited intake of diet due to the time spent travelling to the point of measurement by the sportsmen. One of the most important nutrients for an exercising athlete are carbohydrates [15]. It provides fuel for the brain, central nervous system and enhances performance of prolonged, sustained or intermittent high-intensity exercise [2]. Reduction in carbohydrate stores may result in reduced work rates, impaired skill and concentration and increased perception of effort [2]. Consequently, carbohydrate intake of long-distance race car drivers needs to be improved [28]. The reported protein intake (1.3 ± 0.7 g/kg/day) of our long-distance race car drivers was within the recommendations for athletes although it was lower than that published for other sports [24,27,29]. To support metabolic adaptation, repair, remodeling and protein turnover, current data suggest dietary protein should range form 1.2–2.0 g/kg/day [2]. Therefore, it is important that our study participants consume adequate carbohydrates to spare amino acids for protein synthesis and not waste them for oxidation [2].

According to Thomas et al. [2], the need for some micronutrients increases as exercise stresses many of the metabolic pathways in which micronutrients are required. In the current study, some of the nutrients were above and others were below the recommended dietary allowance for both athletes and the DGE. According to published literature, calcium, vitamin D, the B vitamins, iron, zinc, magnesium, and vitamins C and E are of most concern in athletes. Vitamins and minerals protect cell membranes from damage, and reduce inflammation and muscle soreness during recovery after exercise [2]. Vitamin D regulates calcium, phosphorus absorption and metabolism, plays a key role in maintaining bone health [20] and this might have implications for supporting athletic performance [2]. Calcium intake of 1500 mg/day and 37.5–50 ug/day of vitamin D are needed to optimize bone health in athletes [2]. The low calcium intake recorded is not surprising as it is associated with low energy intake and both were observed in the dietary intake of the athletes [2]. Dietary intake of sodium is usually consumed in the form of salt. It helps to maintain fluid balance, osmotic pressure and contributes to muscle contractions. Sodium also maintains membrane potential, facilitates active transport of molecules across cell membranes as well as water, electrolyte and acid-base balance, and affects extracellular volume [12]. However, high intake is associated with adverse health effects [12]. Therefore, it is important that correction of the deficiency is made in these nutrients for the well-being and success of the athletes. Not reaching the recommendation does not, however, mean the athlete has a deficiency because individual needs must be taken into consideration. Proper hydration is important for optimal health and exercise performance, better concentration and avoidance of fatigue [2]. Athletes are encouraged to consume 5–10 mL/kg body weight of water prior to exercise [2] to stay hydrated. The water intake of the race car drivers was higher than that of mountain runners (2783 ± 1543 mL/day) [24] and above the recommendation for the general adult population of Germany (2600 mL/day). 

### Limitations of Study

The sportsmen spent much time travelling to the venue of their routine medical examination hence it is possible that their normal routine dietary intake was affected. However, the same conditions might be expected while traveling to a competition. It would have been informative to collect additional data on the dietary intake of the sportsmen during a racing activity to compare with the present results. Another limitation of this study is the use of a single 24 h dietary recall. According to Holmes et al. [30] and Jackson et al. [31], 4 and 8 repetitions of 24-h dietary recalls respectively are the most appropriate method for dietary assessment. Nonetheless, the short-term recall as used in the research provides adequate dietary intake data according to Magkos and Yannakoulia [1]. Nevertheless, the current results should be interpreted with caution. Also, a 3-day nutritional protocol may have provided more accurate assessment of the individual athlete’s dietary intake. Due to the unavailability of the race car drivers for 3 days during the period the pilot study was conducted, the 24 h dietary recall was considered appropriate. Direct comparison of our study results with published literature was difficult, since no published data concerning dietary intake of long-distance race car drivers is available. However, this is a strength of our study as we have the first published data on the dietary intake of long-distance race car drivers.

## 5. Conclusions

In conclusion, the results of our pilot study provide a first insight into the dietary intake of long-distance race car drivers and an insight into the development of nutritive recommendations for this target group. The race car drivers in the current study did not meet the daily dietary recommendations according to the DGE and for athletes for both carbohydrate and the majority of the other nutrients measured. Athletes should be supported in achieving their daily dietary requirements to reach their individual recommendations regarding sport-specific training volume and intensity.

## Figures and Tables

**Table 1:** Energy and nutrient intake in comparison with recommendations for athletes and those of the German Nutrition Society (DGE) (mean ± standard deviation (SD)).

Component	Min–Max	Mean	Recommendations for Athletes	Number (n) of Athletes below Minimum Recommendation (n/16)	DGE Recommendations	Number (n) of Athletes below Minimum Recommendation (n/16)
energy intake (kcal/day)	879–4222	2124 ± 814	3600 [15]	14	G	-
**Macronutrients**						
carbohydrate (g/day)	104.9–464.8	236.1 ± 96.7				
carbohydrate (g/kg/day)	1.6–7.5	3.3 ± 1.5	>6 depending on training phase [2]	15	4.7 [16]	15
protein (g/day)	23.5–208.9	95.1 ± 39.4				
protein g/kg/day)	0.4–3.4	1.3 ± 0.7	1.2–2.0 [2]	1	58 [16]	2
fat intake (g/day)	32.11–201.0	85.6 ± 47.1	175.8–1477.7 [2]	8	80 [16]	8
**Vitamins (Vit)**						
vitamin B_1_ (mg/day)	0.5–3.0	1.6 ± 0.8	X	-	1.2 [17]	6
vitamin B_2_ (mg/day)	0.3–2.4	1.3 ± 0.5	X	-	1.4 [17]	9
vitamin B_6_ (mg/day)	0.4–3.5	1.9 ± 0.7	X	-	1.5 [18]	3
vitamin B_12_ (ug/day)	0.4–16.3	6.3 ± 4.3	X	-	3.0 [18]	5
vitamin C (mg/day)	0.8–413.6	161.5 ± 113.0	X	-	91 [19]	5
vitamin D (ug/day)	0.1–5.7	1.8 ± 1.7	20 [2]	16	20 [20]	16
vitamin E (mg/day)	2.9–36.6	12.5 ± 9.5	X	-	14 [18]	13
biotin (ug/day)	9.5–74.7	43.6 ± 17.4	X	-	30–60 [18]	2
folate (ug/day)	414.0–45.4	231.0 ± 90.9	X	-	300 [21]	14
**Minerals**						
sodium (mg/day)	941–5856	2790 ± 1721	X	-	1500 [12]	4
calcium (mg/day)	139–2234	1104 ± 764	1500 [2]	14	1048 [22]	8
magnesium (mg/day)	211–1628	471 ± 335	X	-	350 [18]	5
iron (mg/day)	3.0–28.6	13.2 ± 6.5	X	-	10 [18]	4
zinc (mg/day)	3.6–37.4	13.5 ± 7.7	X	-	10 [18]	6
**Other**						
water (mL/day)	941–7106	3309 ± 1522	2400–3200 [2]	4	2600 [16]	5
fiber (g/day)	6.2–44.2	21.4 ± 9.4	X	-	30 [18]	2
alcohol (g/day)	0.0–9.9	0.8 ± 2.5	X	-	20 (maximum allowed amount) [18]	0

Legend: X (there are no recommended dietary levels for athletes); G (guiding values are available which is 3000 kcal/day [18]).

## Appendix A

Structured 24 h diet recall questionnaire

Now I’d like to know what you have eaten during the last 24 h. Try to remember and describe as precise as possible. Let’s start with the breakfast. (Take notes as precise as possible!)

- What have you eaten for breakfast? What did you drink?
- Did you have a snack before lunch? Did you drink something? If yes, what?
- What did you eat for lunch? What did you drink?
- Did you have another snack before dinner?
- What did you eat for dinner? What did you drink?
- Did you eat or drink something after dinner before going to bed?
